# Synthesis and Modification of Tetrahedron Li_10.35_Si_1.35_P_1.65_S_12_ *via* Elemental Doping for All-Solid-State Lithium Batteries

**DOI:** 10.3389/fchem.2022.851264

**Published:** 2022-03-22

**Authors:** Yuanzhong Lin, Jian Chen, Jiawei Yan, Yanhua Zhuang, Hengyi Lu, Chenyang Zhao

**Affiliations:** ^1^ College of Chemistry and Environmental Engineering, Shenzhen University, Shenzhen, China; ^2^ Walker Department of Mechanical Engineering, The University of Texas at Austin, Austin, TX, United States

**Keywords:** sulfide solid-state electrolyte, solid-state battery, Li_10.35_Si_1.35_P_1.65_S_12_, ionic conductivity, cation substitution

## Abstract

Solid-state electrolyte (SSE), as the core component of solid-state batteries, plays a critical role in the performance of the batteries. Currently, the development of SSE is still hindered by its high price, low ionic conductivity, and poor interface stability. In this work, we report the tailored synthesis of a high ionic conductive and low cost sulfide SSE for all-solid-state lithium batteries. The Li_10.35_Si_1.35_P_1.65_S_12_ with favorable tetragonal structure was synthesis by increasing the concentration of Si^4+^, which shows an ionic conductivity of 4.28 × 10^−3^ S cm^−1^ and a wide electrochemical stability window of up to 5 V. By further modifying the composition of the electrolyte *via* ionic doping, the ionic conductivity of Li_10.35_Si_1.35_P_1.65_S_12_ can be further enhanced. Among them, the 1% Co^4+^-doped Li_10.35_Si_1.35_P_1.65_S_12_ shows the highest ionic conductivity of 6.91 × 10^−3^ S cm^−1^, 40% higher than the undoped one. This can be attributed to the broadened MS_4_^−^ tetrahedrons and increased Li^+^ concentration. As a demonstration, an all-solid-state Li metal battery was assembled using TiS_2_ as the cathode and 1% Co^4+^-doped Li_10.35_Si_1.35_P_1.65_S_12_ as the electrolyte, showing capacity retention of 72% at the 110th cycle. This strategy is simple and can be easily extended for the construction of other high-performance sulfide SSEs.

## Introduction

With the gradual popularity of electric vehicles, the safety issues of lithium-ion batteries and mileage anxiety have become the biggest obstacles that hinder their further development (Chen et al., 2021). Regarding these, solid-state batteries are considered as one of the most promising solutions. On the one hand, by using solid-state electrolyte (SSE), batteries can reduce or even completely avoid the use of flammable organic liquid electrolyte, which can effectively avoid the spontaneous combustion of batteries in special circumstances (Bates et al., 1992; Dudney et al., 1992; Manthiram et al., 2017); on the other hand, solid-state batteries can use lithium metal and high-voltage cathode materials as electrode materials, which increases the energy density of batteries and reduces mileage anxiety (De Luna et al., 2021). At present, the SSE still cannot meet the requirements of commercial applications. The major problems are low ion conductivity, high production cost, and poor stability with electrodes (Porz et al., 2017; Kim et al., 2021).

Since the discovery of 2011, Li_10_GeP_2_S_12_ (LGPS) SSE has attracted increasing attention due to its extremely high ionic conductivity, which is comparable to commercial liquid electrolyte (Kanno and Murayama, 2001; Kamaya et al., 2011). However, its high production cost and poor stability with lithium metal restrict its large-scale applications (Wenzel et al., 2016; Chen et al., 2017; Madsen et al., 2020). As the same main group of Ge, Si is the second rich element in the Earth’s crust. The replacement of Si to Ge will significantly reduce the cost of the electrolyte. More importantly, it is predicted that Si-substituted electrolyte (LiSiPS) has the same body-centered cubic-like anion framework as LGPS, which allows fast Li^+^ hops along the c-axis (Wang et al., 2015; Harm et al., 2019). However, the preparation of phase-pure LGPS-type Li_10_SiP_2_S_12_ was not successful due to the small ionic radius of Si^4+^ (Ong et al., 2013; Kuhn et al., 2014; Zhang et al., 2019). The ionic conductivity of LiSiPS is far lower than expected and needs to be further enhanced for battery applications (Kato et al., 2014; Kuhn et al., 2014; Kato et al., 2016; Sun et al., 2017; Chen et al., 2018; Wu et al., 2018).

In the LGPS-type electrolyte, the MS_4_ tetrahedron center is occupied by metal cations and P^5+^ (De Luna et al., 2021). The radii of cations directly affect the size of the tetrahedron and the crystal structure stability. Because of the smaller radius of Si^4+^ (0.026 nm) compared to Ge^4+^ (0.039 nm), the structure of Li_10_SiP_2_S_12_ changes to orthorhombic, which is less favorable for Li^+^ transport (Shannon, 1976). On one hand, increasing the concentration of Si^4+^ can partially compensate the volume loss [P^5+^ (0.017 nm) is replaced by Si^4+^ (0.026 nm)] and is believed to enhance the stability of LiSiPS (Kondo et al., 1992; Murayama et al., 2002; Whiteley et al., 2014). The non-equivalent substitution can also increase the concentration of Li^+^ due to charge compensation, which is beneficial for Li^+^ transport. On the other, the introduction of other metal ions with ionic radius close to Ge^4+^ will largely expand the size of the tetrahedrons and reduce the Li^+^ transport resistance. By further modifying the composition of the ionic framework, the interaction between Li^+^ and the host may be even weakened (Adams and Rao, 2012; Bron et al., 2016; Kim and Martin, 2019).

On the basis of the above assumption, in this work, a low-cost and high ionic conductive LGPS-type LiSiPS was prepared through composition regulation and doping. The Li_10.35_Si_1.35_P_1.65_S_12_ obtained shows high ionic conductivity of 4.28 × 10^−3^ S cm^−1^ at room temperature and good electrochemical stability up to 5 V. Doping Li_10.35_Si_1.35_P_1.65_S_12_ with Co^4+^, which is equivalent to Si^4+^ but has a larger radius than Si^4+^, broadens the MS_4_ tetrahedrons and reduces the Li^+^ transmission resistance. The 1% Co^4+^-doped Li_10.35_Si_1.35_P_1.65_S_12_ shows the best electrochemical performances. It has an ionic conductivity of 6.91 × 10^−3^ S cm^−1^ and a Li^+^ migration number as high as 0.97. The ion conductivity is about 40% higher than that of undoped sample. The assembled Li/SSE/TiS_2_ all-solid-state lithium battery shows stable lifespan of more than 100 cycles with a specific discharge capacity of 70 mAh g^−1^ (Li et al., 2016). Finally, to verify the effect of anion doping on the performance of the SSE, Se^2−^, which has a larger radius than S^2−^, was introduced and a CoSe_2_-doped LiSiPS sulfide SSE was prepared. The enhanced ionic conductivity can be attributed to the large ion radius of Se^2−^, which widens the migration channel for Li^+^, and the larger polarization characteristics of Se^2−^ that effectively reduce the affinity to Li^+^.

## Experimental Section

### Materials Synthesis

The LiSiPS SSEs were synthesized by high-energy ball milling process followed by thermal annealing. Stoichiometric proportion of Li_2_S, SiS_2_ and P_2_S_5_ and 10 zirconia balls (10 mm in diameter) were added to the ball mill tank in an Ar-filled glove box. The mixture was ball milled for 40 h at 500 rpm. A 15-min rest was set after 15-min ball milling to avoid overheat of the machine. The ground powder was put into a quartz tube and sealed under vacuum. The LiSiPS SSEs were finally obtained by heat treatment at 400°C–500°C for 3 days and naturally cooled to room temperature. The modification of the LiSiPS SSEs was carried out under the same conditions except the addition of a certain amount of CoS_2_, TiS_2_, and CoSe_2_. The doping ratio (0%, 0.5%, 1%, 2%, and 3%) refers to the proportion of M^4+^:Si^4+^ (M = Si^4+^, Ti^4+^).

### Materials Characterization

The crystal structure was characterized by X-ray diffraction (XRD; D8ADVANCE) with a Cu Kα source between 10° and 80°. The morphology and microstructure of the samples were characterized by scanning electron microscope (SEM; JSM-7800F) with acceleration voltage of 30 kV. The molecular vibrations and rotations were characterized by Raman spectra with a wavelength of 532 nm in range of 200–4,000 cm^−1^. The surface elements and chemical states of the samples were characterized by X-ray photoelectron spectroscopy (XPS; K-Alpha+).

### Electrochemical Measurement

The ionic conductivity was measured by sandwiching the electrolyte pellets between two stainless steel sheets after hot-pressed at 380 MPa at 250°C. Electrochemical impedance spectroscopy (EIS) at frequencies from 1 MHz to 10 Hz was performed on the blocking cells with amplitude of 10 mV. The Li^+^ migration number (*t*_*Li+*_) was measured by Li/SSE/Li symmetric cell based on the following equation:

$$t_{Li^{+}}=\frac{I_{ss}R_{\text{bss}}\left(V-I_{0}R_{i0}\right)}{I_{0}R_{\text{b}0}\left(V-I_{ss}R_{iss}\right)}$$ (1)

where *V* is the voltage applied; *I*_0_ is the initial current; *R*_*b0*_ is the initial bulk resistance of SSE; *R*_*i0*_ is the initial resistance of the passivation layer; and *I*_ss_, *R*_*bss*_, and *R*_*iss*_ are the current, bulk resistance of SSE, and the resistance of the passivation layer at steady state, respectively. The chemical stability to Li metal was measured using the same symmetric cell. The current density and duration time were set as 0.05 mA cm^−2^ and 1 h, respectively. The electrochemical window of electrolyte was estimated by Cyclic Voltammetry (CV) with stainless steel sheet and Li foil as the electrodes. The sample was scanned between −0.5 and 5 V with a scan rate of 1 mV s^−1^.

### Electrode Preparation and All-Solid-State Battery Assembly

To avoid the direct contact between the sulfide SSE and lithium metal, a fluorination of lithium metal was conducted (Fan et al., 2018). A certain amount of Lithiumbis (fluorosulfonyl)imide was added to anhydrous DME to get a 6 mol L^−1^ LiFSI-DME solution. The solution was then dropped on both sides of the lithium metal. The 10-mm lithium foil was then placed on the surface of the SSE pellets and dried overnight in a vacuum oven at 120°C. The cathode was prepared by mixing TiS_2_, acetylene black, and SSE with a mass ratio of 5:1:4. They were first manually mixed in a mortar for 10 min and then ball-milled for 6 h at 500 rpm. The powder sample after ball milling was used as composite cathode material for all-solid-state lithium battery.

For the battery assembly, 60-mg SSE was added into a custom mold and pressed into pellets at 250 MPa. Then, the composite cathode material was added to the upper side of the electrolyte and pressed under a pressure of 375 MPa for 5 min. The areal density of TiS_2_ was ca. 4 mg cm^−2^. After compaction, the fluorinated lithium metal was put onto the other side of the SSE. The battery was cycled between 1.5 and 3 V at 0.1 C.

## Results and Discussion

### Structure and Property of the Li_10.35_Si_1.35_P_1.65_S_12_ SSE

**FIGURE 1:**
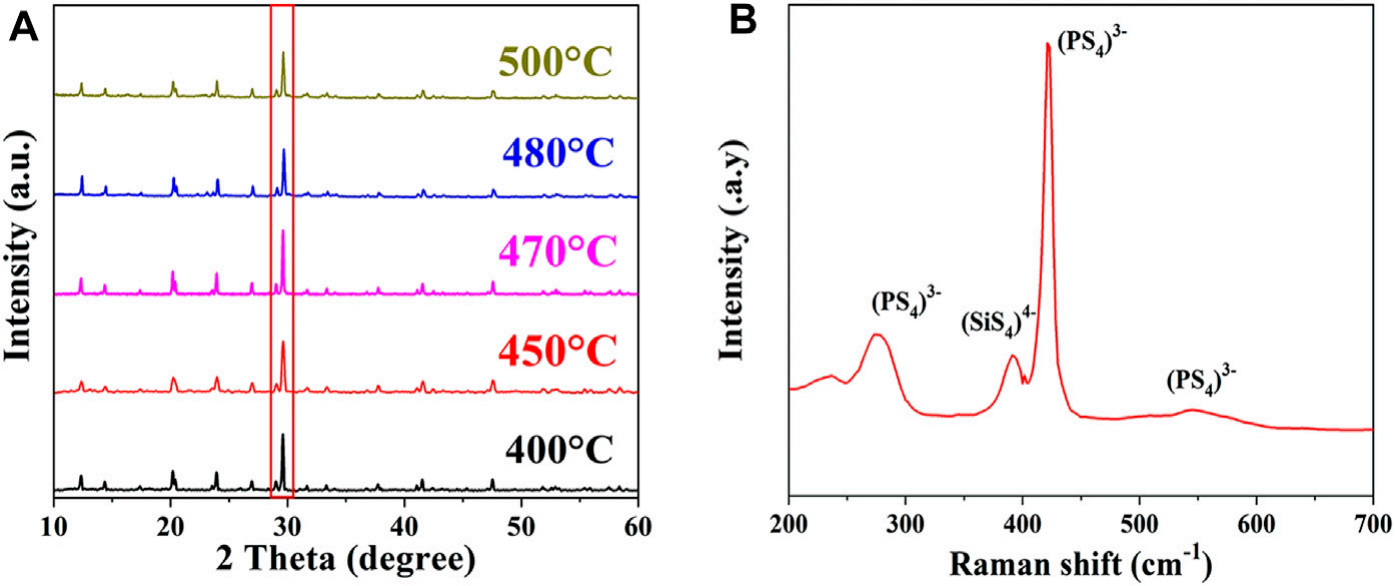
XRD patterns of Li_10.35_Si_1.35_P_1.65_S_12_ synthesized at different temperatures **(A)** and typical Raman spectrum of the Li_10.35_Si_1.35_P_1.65_S_12_ **(B)**.

The structure of the synthesized Li_10.35_Si_1.35_P_1.65_S_12_ SSE was first analyzed by XRD and Raman. The normalized XRD patterns are shown in Figure 1A. All the diffraction peaks are in good consistent with LGPS, indicating the successful synthesis of phase-pure tetragonal Li_10.35_Si_1.35_P_1.65_S_12_ with a space group of P42/nmc (137). Compared to LGPS, the Li_10.35_Si_1.35_P_1.65_S_12_ has higher content of tetravalent center ions, which enhances the stability of the body-centered cubic-like anion framework (Kuhn et al., 2014). The sample synthesized between 400°C and 500°C exhibits identical XRD patterns. Among them, the sample at 470°C shows the highest crystallinity. The Raman spectrum of the Li_10.35_Si_1.35_P_1.65_S_12_ (470°C) is shown in Figure 1B. The peaks at 275, 420, 550, and 575 cm^−1^ are attributed to the characteristic signals of PS_4_^3−^, whereas the peak at 390 cm^−1^ comes from the stretching vibration of Si-S^−^ bond in SiS_4_^4-^(Pradel and Ribes, 1989). The above results confirmed the successful synthesis of the desirable LGPS-type Li_10.35_Si_1.35_P_1.65_S_12_ SSE.

**FIGURE 2:**
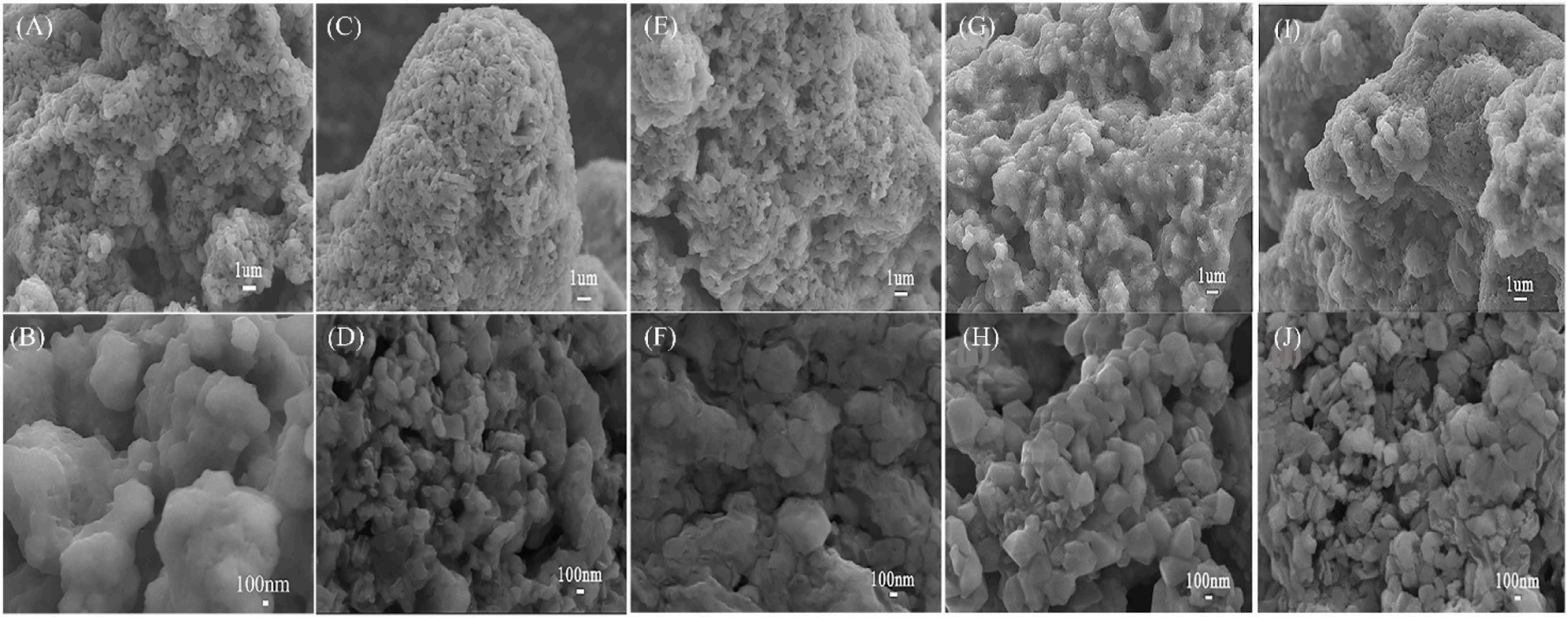
SEM images of Li_10.35_Si_1.35_P_1.65_S_12_ synthesized at 400°C **(A,B)**, 450°C **(C,D)**, 470°C **(E,F)**, 480°C **(G,H)**, and 500°C **(I,J)**.

**FIGURE 3:**
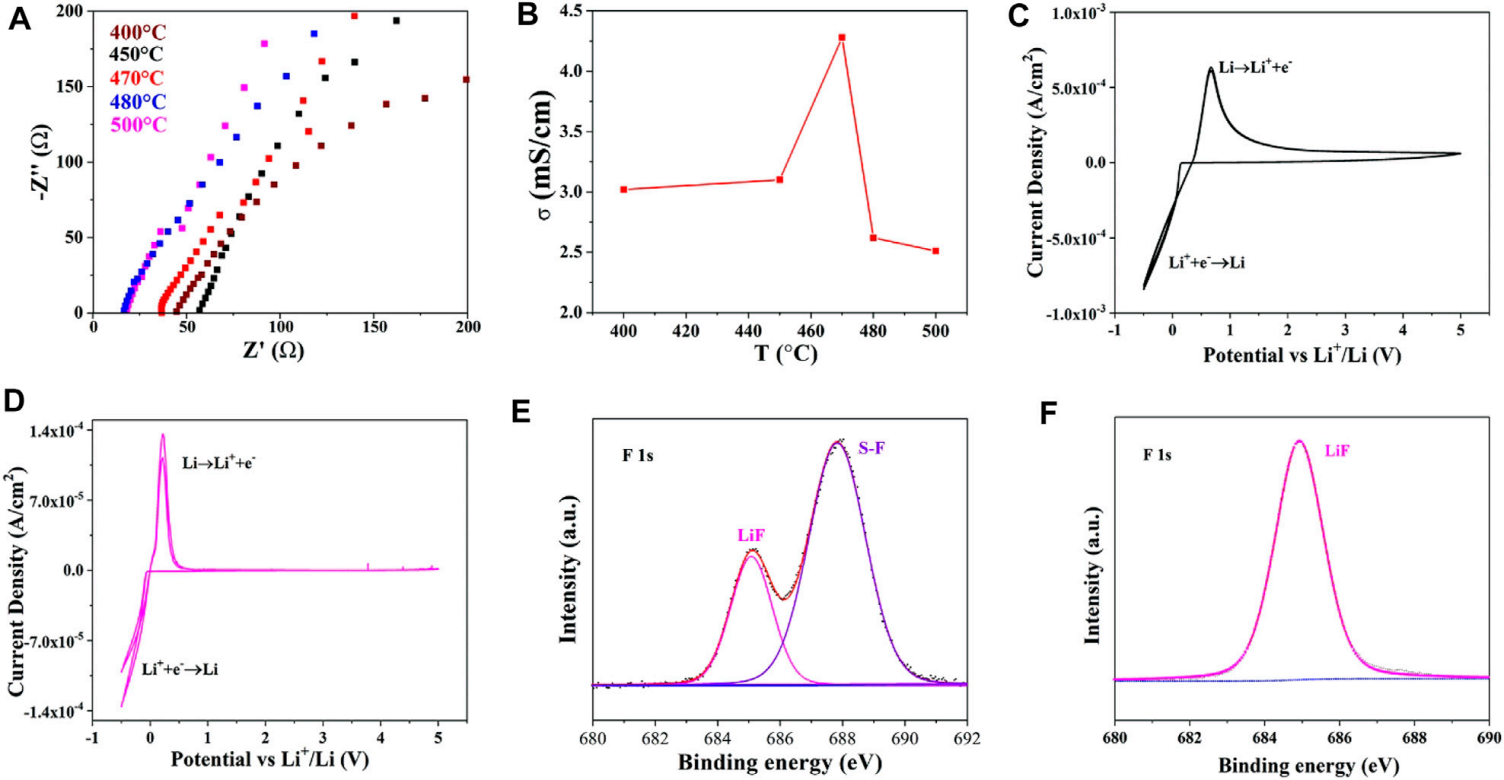
Nyquist plots of Li_10.35_Si_1.35_P_1.65_S_12_ synthesized at different temperatures **(A)**, the corresponding ionic conductivities **(B)**, CV test of Li_10.35_Si_1.35_P_1.65_S_12_ with pristine lithium metal **(C)** and modified lithium metal **(D)** as electrodes, XPS of lithium metal before **(E)** and after **(F)** polarization test.

The morphologies of Li_10.35_Si_1.35_P_1.65_S_12_ synthesized at different temperatures are shown in Figure 2. All the electrolyte powders are composed of micron-sized granules with irregular shape. From the high-resolution view, it can be seen that the electrolyte synthesized at 470°C has higher degree of particle agglomerations. This is beneficial to reduce the interface impedance of the electrolyte and promote Li^+^ transfer across the grain boundaries. The ionic conductivity of Li_10.35_Si_1.35_P_1.65_S_12_ was then measured by EIS. To lower the interface impedance inside the electrolyte, the samples were hot presses to make the power more compact and denser. The impedances of Li_10.35_Si_1.35_P_1.65_S_12_ synthesized at 470°C were tested under different temperature and pressure, and the results are shown in Supplementary Figure S8. As shown in Supplementary Figure S8, the sample treated at 375 MPa and 250°C shows the lowest resistance. Therefore, they are chosen as the optimized hot pressing parameters unless otherwise specified. Figure 3A shows the Nyquist plots of the samples.

The detailed information is shown in Supplementary Table S1. As shown in Figure 3A, all the plots show the diagonal characteristics, indicating the ionic conductor nature of the electrolytes. The negligible semicircles at high-frequency region signify small grain boundary impedance of the electrolytes. The ionic conductivity of Li_10.35_Si_1.35_P_1.65_S_12_ increases first with the annealing temperature due to the enhanced crystallinity and reaches 4.28 × 10^−3^ S cm^−1^ at 470°C (Figure 3B). However, the ionic conductivity decreases with the further increase of the temperature. This may be attributed to the slight decomposition of Li_10.35_Si_1.35_P_1.65_S_12_, as indicated by the reduced peak intensity in XRD.

The electrochemical window of Li_10.35_Si_1.35_P_1.65_S_12_ (470°C) was investigated by CV. An asymmetric configuration was adopted using stainless steel and lithium metal as electrodes. As shown in Figure 3C, the sharp peak started at 0 V can be attributed to the Li^+^ deposition on the lithium metal surface. Its dissolution is found at 0.5 V during the anodic scan. However, the anodic current lasts throughout the test window, indicating a continuous interface reaction between Li and Li_10.35_Si_1.35_P_1.65_S_12_. To avoid this, a thin layer of LiF was *in situ* deposited to prevent the direct contact between them (details seen in the experimental section) (Porz et al., 2017). The XPS spectrum of the modified lithium is shown in Figure 3E. The characteristic peak at 685 eV is assigned to LiF, confirming the successful formation of LiF protection layer. The peak that appears at 687.8 eV corresponds to S-F bonds of LiFSI precursor, which further reacts with Li and converts to LiF during the following electrochemical reaction (Figure 3F). As a result, the interface side reaction is largely suppressed. As shown in Figure 3D, only one pair of redox peaks corresponding to $L{\text{i}}^{+}+e^{-}↔Li$ is observed. The Li_10.35_Si_1.35_P_1.65_S_12_ is stable up to 5 V, showing potential to match with high voltage cathodes. In the following text, the Li metals are all modified using the same method unless otherwise specified.

**FIGURE 4:**
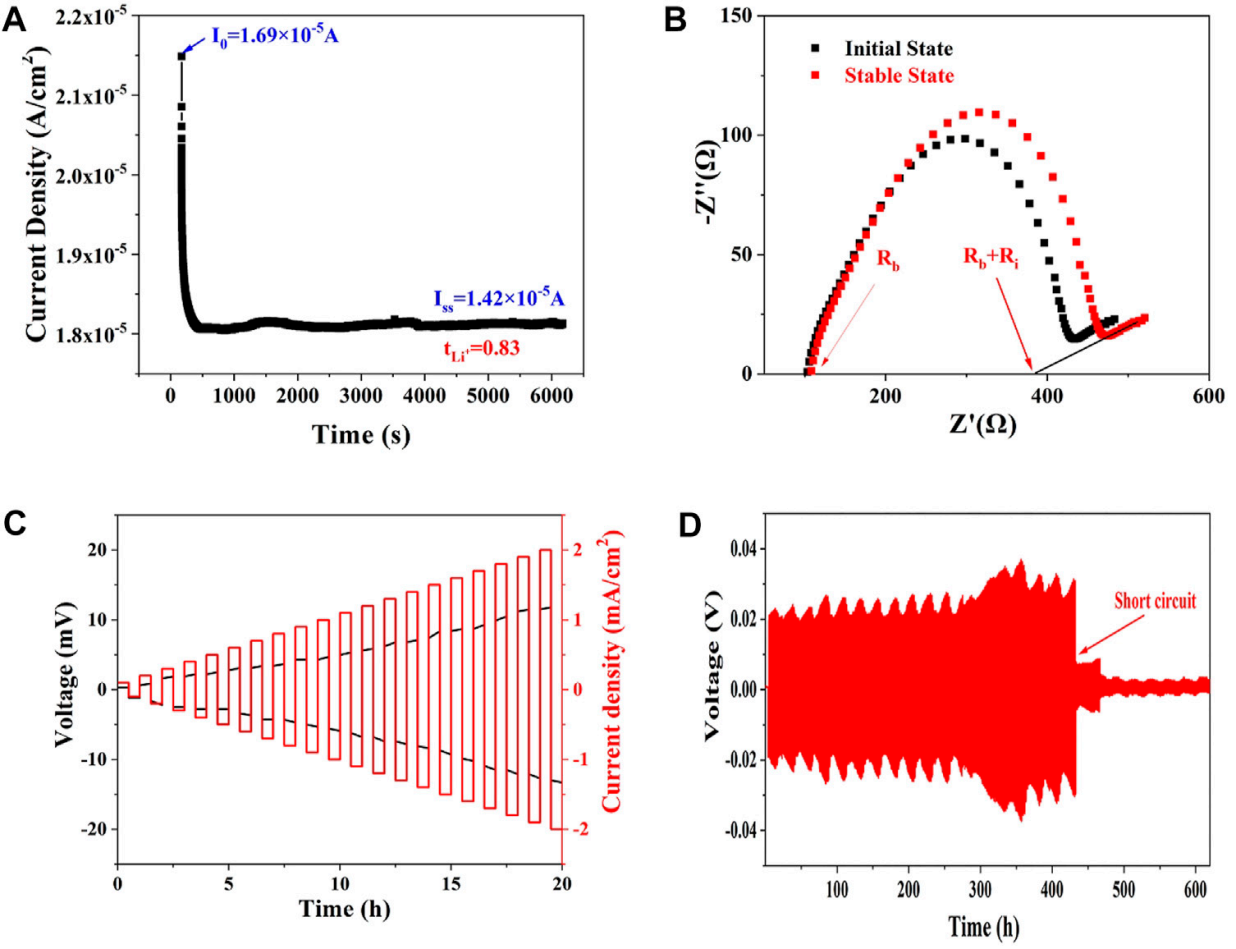
The time-current curve of Li/SSE/Li symmetric cell under 10 mV **(A)**, the Nyquist plots of the cell at initial and steady states **(B)**, and polarization curves of the Li/SSE/Li symmetric cell under different current densities **(C,D)**.

The ionic migration number of Li_10.35_Si_1.35_P_1.65_S_12_ (470°C) was then measured to confirm the proportion of Li^+^ transport in the electrolyte. As shown in Figures 4A,B, the total impedance increases from 363.4 to 384.2 Ω due to the space charge separation at the interface. The polarization current decreases from initial 1.69 × 10^−5^ A to steady state 1.42 × 10^−5^ A. The ionic migration number was calculated to be 0.83 according to Eq. 1. The high ionic migration number explains the high ionic conductivity of Li_10.35_Si_1.35_P_1.65_S_12_, especially compared with polymer and oxide SSEs. The ability of Li_10.35_Si_1.35_P_1.65_S_12_ to inhibit the growth of Li dendrites was evaluated using Li/SSE/Li symmetrical cell. As shown in Figure 4C, with the increase of current density from 0.1 mA cm^−2^ to 2 mA cm^−2^, the overpotential increases from 0.3 to 13.3 mV without short circuit. This result indicates that the limiting current density of Li_10.35_Si_1.35_P_1.65_S_12_ is higher than 2 mA cm^−2^. Figure 4D shows the constant-current polarization curve at an areal capacity of 0.05 mAh cm^−2^. The cell is stable up to 432 h, showing good resistance to the growth of lithium dendrites.

### Modification of the Li_10.35_Si_1.35_P_1.65_S_12_ SSE *via* Cation Doping

To further enhance the ionic conductivity of Li_10.35_Si_1.35_P_1.65_S_12_, Ti^4+^ (0.042 nm) and Co^4+^ (0.04 nm) were introduced into the lattice of Li_10.35_Si_1.35_P_1.65_S_12_ because of their comparable ionic radii with Ge^4+^ but much lower price. The XRD patterns of the synthesized CoS_2_ and TiS_2_ are shown in Supplementary Figure S1. After ball milling and thermal annealing, Co^4+^ and Ti4^+^-doped Li_10.35_Si_1.35_P_1.65_S_12_ SSE were obtained.

**FIGURE 5:**
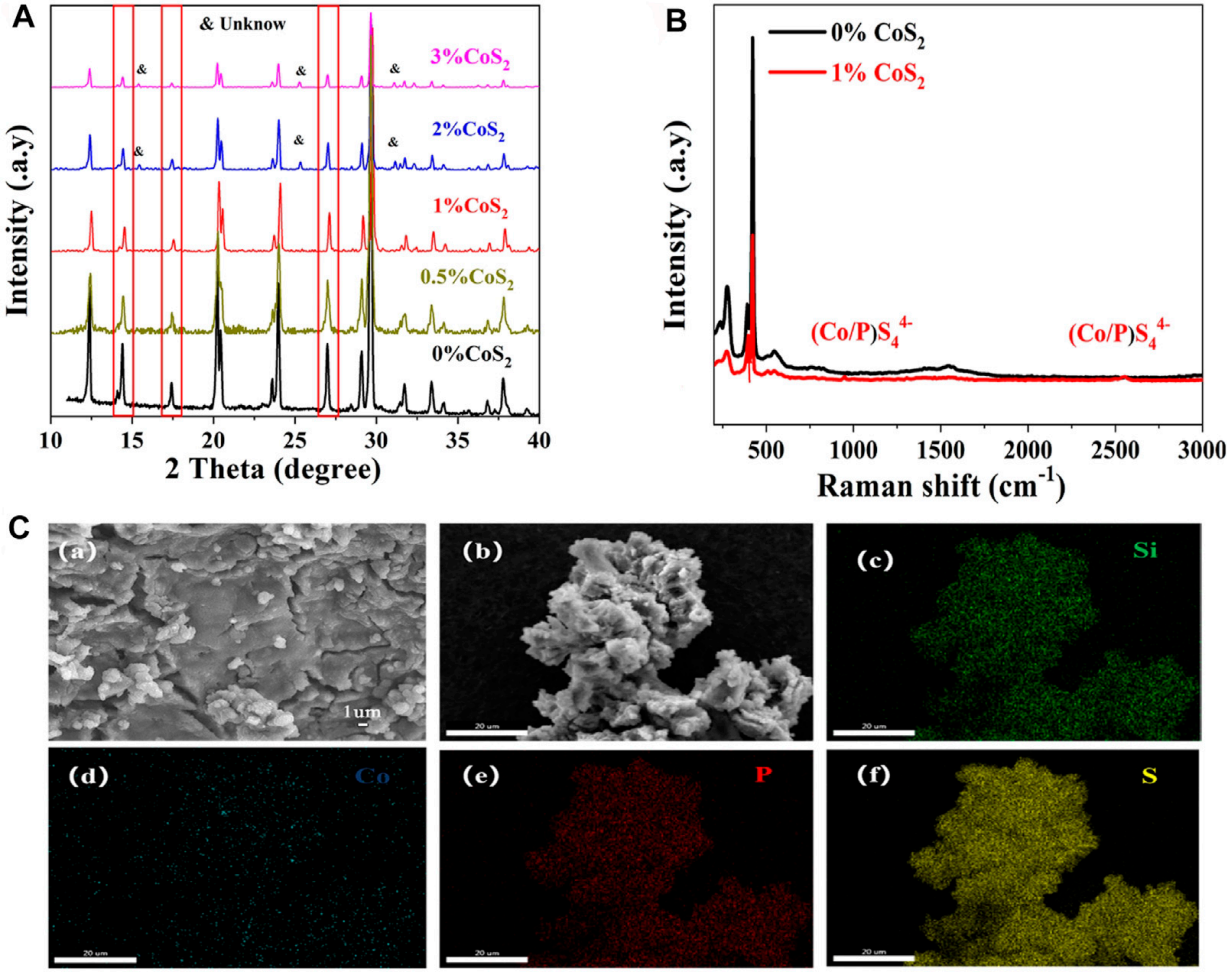
XRD patterns of Li_10.35_Si_1.35-x_Co_x_P_1.65_S_12_ with different Co^4+^ doping ratios **(A)**, Raman Spectra of pristine and 1% Co^4+^-doped electrolytes **(B)**, and SEM and EDS images of 1% Co^4+^-doped Li_10.35_Si_1.35-x_Co_x_P_1.65_S_12_ **(C)**.

Figure 5A shows the normalized XRD patterns of Co4^+^-doped Li_10.35_Si_1.35_P_1.65_S_12_. The position of the peaks match well with Li_10.35_Si_1.35_P_1.65_S_12_, indicating that the doping of Co^4+^ does not change the initial tetragonal structure. The crystallinity decreases with the increase of Co^4+^ content, showing more defects are introduced which can promote the transport of Li^+^. Unknown impurity appears (15.5°, 25.3°, and 32.3°) when the doping ratio of Co^4+^ is higher than 2%. The higher content of Co^4+^ exceeds the limit of the solid solution and leads to the emergence of new phase. It worth to note that peak shifts to higher angles are observed (14.4°, 17.4°, and 20.2°) at relatively low doping ratios (0.5% and 1%). This indicates a constriction of crystal lattice and contradicts with the previous prediction. To explore the crystal parameters after doping, the Rietveld refinement for 1% Co^4+^-doped Li_10.35_Si_1.35_P_1.65_S_12_ was carried out. The results are shown in Supplementary Table S2 and Supplementary Figure S2. The 1% Co4^+^-doped Li_10.35_Si_1.35_P_1.65_S_12_ shows a P4_2_/nmc space group with lattice constants of *a* = 8.6731 Å and *c* = 12.5331 Å. Compared to original Li_10.35_Si_1.35_P_1.65_S_12_ (*a* = 8.6708 Å and *c* = 12.5396 Å), the unit cell expands along the *a*-direction but shrinks slightly in the *c*-direction due to the Co^4+^ doping, resulting in an expansion of the cell volume. This broadens the channels of Li^+^ transport, which further enhances the ionic conductivity of electrolytes. The XRD patterns of Ti^4+^-doped Li_10.35_Si_1.35_P_1.65_S_12_ show similarly characteristics (Supplementary Figure S3). The Raman spectrum of 1% Co-doped Li_10.35_Si_1.35_P_1.65_S_124_ is shown in Figure 5B. Compared to Li_10.35_Si_1.35_P_1.65_S_12_, two new peaks emerge at 950 and 2,550 cm^−1^, which are related to (Co./P)S_4_^4−^ structure. The morphology of the 1% Co^4+^-doped Li_10.35_Si_1.35_P_1.65_S_12_ is shown in Figure 5C. It can be seen that most of the electrolyte granules melt together during the thermal treatment, and some are clearly visible on the surface. All the elements are uniformly distributed within the electrolyte, again confirming the successful doping of Co^4+^.

**FIGURE 6:**
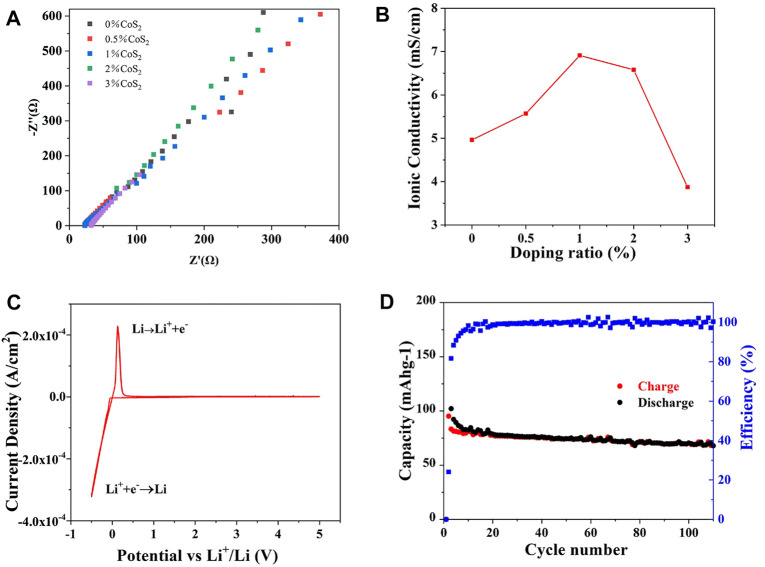
Ionic conductivity of Co^4+^-doped Li_10.35_Si_1.35_P_1.65_S_12_ **(A,B)**, CV curve of 1% Co^4+^-doped electrolyte **(C)**, and cycling stability of the all-solid-state lithium battery **(D)**.

The ionic conductivity of Co^4+^-doped Li_10.35_Si_1.35_P_1.65_S_12_ was measured by blocking cell. The testing parameters are summarized in Supplementary Table S3. As shown in Figures 6A,B, the ionic conductivity of Li_10.35_Si_1.35_P_1.65_S_12_ is effectively enhanced by Co^4+^ doping, and 1% Co^4+^-doped sample shows the highest ionic conductivity of 6.91 mS cm^−1^, 40% higher than the undoped one. The enhanced ionic conductivity could be attributed to the LGPS-type anion packing, the higher concentration of Li^+^ induced by M^4+^ substitution, and the broadened Li^+^ transport path. Compared to Co^4+^, the doping of Ti^4+^ can also enhance the ionic conductivity of Li_10.35_Si_1.35_P_1.65_S_12_ by 15% (5.69 mS cm^−1^) when 0.5% TiS_2_ was added, showing the effectiveness of the doping strategy (Suplementary Figure S4). The slightly difference may be attributed to the limited doping ratio of Ti^4+^. The electrochemical windows of the Co^4+^-doped samples were studied using asymmetrical cell. Figure 6C and Suplementary Figure S5 show the CV curves of 0.5%, 1%, and 2% Co^4+^-doped Li_10.35_Si_1.35_P_1.65_S_12_. Only one pair of redox peak at around 0 V is observed for all the samples, indicating that they are electrochemically stable between 0 and 5 V. The ionic migration number of 1% Co^4+^-doped Li_10.35_Si_1.35_P_1.65_S_12_ is calculated to be 0.97. The much increased value shows that Co^4+^ doping can promote the migration of Li^+^. As a demonstration, a solid-state Li battery was assembled using the 1% Co^4+^-doped Li_10.35_Si_1.35_P_1.65_S_12_ as electrolyte and TiS_2_ as the active material. As shown in Figure 6D, the specific charge capacity of the first cycle is 95.2 mAh g^−1^, and 72% of the initial capacity can be retained after 110 cycles, showing stable performance in practical use. The low Coulombic efficiency of the first cycle can be attributed to the decomposition of LiFSI precursor, as shown in Figure 3F.

### Further Attempt for the Performance Improvement of Li_10.35_Si_1.35_P_1.65_S_12_

The above results have inspired us to further improve the ionic conductivity of Li_10.35_Si_1.35_P_1.65_S_12_ through dual doping. The Se^2−^ (0.198 nm) has a large ionic radius than S^2−^ (0.184 nm). Similar to Co^4+^, the proper Se^2−^ anion substitution may also enlarge the diffusion channel of Li^+^. What is more, the higher polarizability of Se^2−^ can further weaken the binding between Li^+^ and the anion framework, thus improving the ionic conductivity of Li_10.35_Si_1.35_P_1.65_S_12_.

**FIGURE 7:**
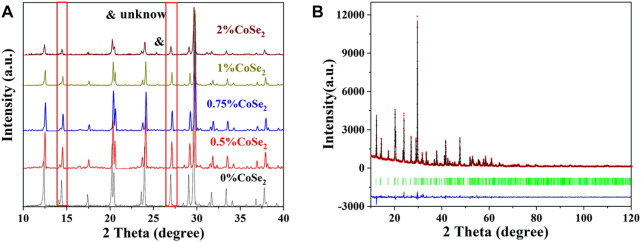
XRD pattern of the CoSe_2_ doping Li_10.35_Si_1.35_P_1.65_S_12_ **(A)** and the Rietveld refinement of 1% CoSe_2_-doped sample **(B)**.

CoSe_2_ was first synthesized by solid-phase reaction. The CoSe_2_ consists of cubic-shaped particles with size of ca. 1 µm (Supplementary Figure S6), and its XRD is shown in Supplementary Figure S7. After ball milling and thermal treatment, sintered irregular particles are obtained. The normalized XRD patterns of CoSe_2_-doped Li_10.35_Si_1.35_P_1.65_S_12_ SSEs are shown in Figure 7A. All the samples exhibit the same LGPS-type structure. With the increase of doping ratio, the peak intensity decreases gradually and some peaks shift to lower angles, similar to the case of CoS_2_. When the doping ratio of CoSe_2_ exceeds 1%, the saturation of CoSe_2_ is reached and impurity phase appears at 26°. The cell parameters of the 1% CoSe_2_-doped Li_10.35_Si_1.35_P_1.65_S_12_ were analyzed by Rietveld refinement. The results are shown in Figure 7B and Supplementary Table S4. The dual-doped sample maintains the same tetragonal structure. The lattice parameters are a = 8.677 Å and c = 12.535 Å with a cell volume of 943.835 Å^3^. These values are larger than that of 1% Co4^+^-doped sample, indicating the successful doping of Co^4+^ and Se^2−^ into the lattice of Li_10.35_Si_1.35_P_1.65_S_12_. The incorporation of Co^4+^ and Se^2−^ mainly induces the lattice expansion along the *a*-axis.

**FIGURE 8:**
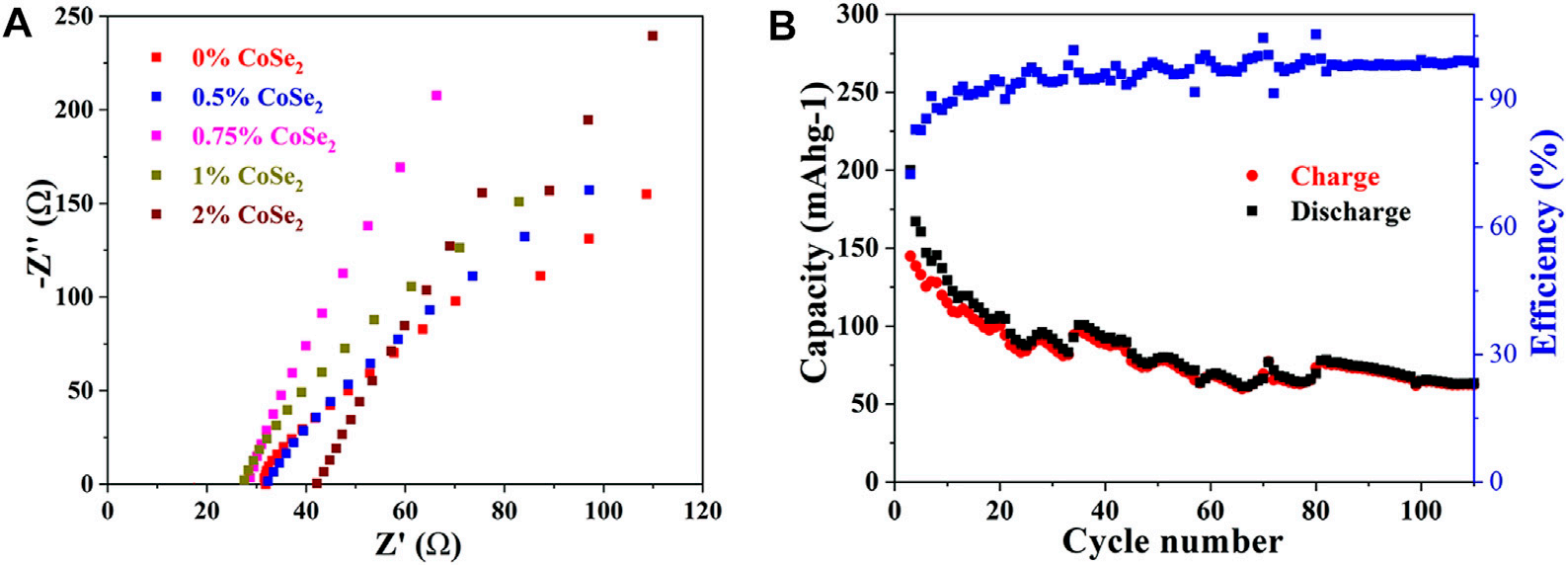
**(A)** Nyquist plots of CoSe_2_-doped Li_10.35_Si_1.35_P_1.65_S_12_ **(B)** Cycling stability of the all-solid-state lithium battery assembled with 1% CoSe_2_ doped SSE.

The ionic conductivities of the CoSe_2_-doped samples were measured by EIS. As shown in Figure 8A, all the samples show negligible grain boundary resistances. With the increase of CoSe_2_ doping ratio, the impedance first decreases and then increases, and 1% CoSe_2_-doped sample has the highest ionic conductivity of 6.07 × 10^−3^ S cm^−1^, slight lower than that of 1% Co^4+^-doped one. The electronic conductivity of the 1% CoSe2-doped sample was added in Supplementary Figure S9. The electronic conductivity of 1% CoSe_2_-doped electrolyte was measured to be 1.89 × 10^−7^ S cm^−1^, four orders of magnitude lower than its ionic conductivity. The results show that the doping of Co and Se in the LSiPS system can greatly enhance the ionic conductivity. The ionic conductivity obtained in this work is comparable or even better than the previous reports as shown in Supplementary Table S5.

An all-solid-state Li battery was then assembled using the 1% CoSe_2_-doped sample as SSE. The cell shows an initial specific charge capacity 160 mAh g^−1^ and the reversible specific capacity decreases gradually to 65 mAh g^−1^ at the end of 100th (Figure 8B). This result shows that, although the incorporation of Se^2+^ can enlarge the MS_4-x_^2−^/Se_x_^2^^−^ tetrahedrons, the conductivity of doped Li_10.35_Si_1.35_P_1.65_S_12_ strongly related to its purity and composition. Further research is still underway and will be reported elsewhere.

## Conclusion

In summary, a low-cost and high-quality sulfide SSE Li_10.35_Si_1.35_P_1.65_S_12_ with favorable tetrahedron structure was synthesized, which was further modified by elemental doping. The 1% Co^4+^-doped Li_10.35_Si_1.35_P_1.65_S_12_ maintained body-centered cubic-like anion framework and shows a high ionic conductivity of 6.91 × 10^−3^ S cm^−1^ due to easy Li^+^ transport between enlarged tetrahedral sites. A high Li^+^ transport number of 0.97 and wide electrochemical stability window of up to 5 V are also reached. These interesting characteristics endow the sample with good electrochemical performance when assembled into all-solid-state Li batteries.

## Data Availability

The original contributions presented in the study are included in the article/Supplementary Material; further inquiries can be directed to the corresponding author.
