# Supplementary material for: Synthesis and Modification of Tetrahedron Li10.35Si1.35P1.65S12 via Elemental Doping for All-Solid-State Lithium Batteries
Source: Front Chem. 2022 Mar 22;10:851264. doi: 10.3389/fchem.2022.851264 (PMC8980264; doi:10.3389/fchem.2022.851264)
Supplement: Supplementary file 1 [file DataSheet1.docx]

Synthesis and Modification of Tetrahedron Li_10.35_Si_1.35_P_1.65_S_12_ via Elemental Doping for All-solid-state Lithium Batteries

Yuanzhong Lin, Jian Chen, Jiawei Yan, Yanhua Zhuang, Chenyang Zhao^*^

College of chemistry and environmental engineering, Shenzhen University, Shenzhen, Guangdong, China


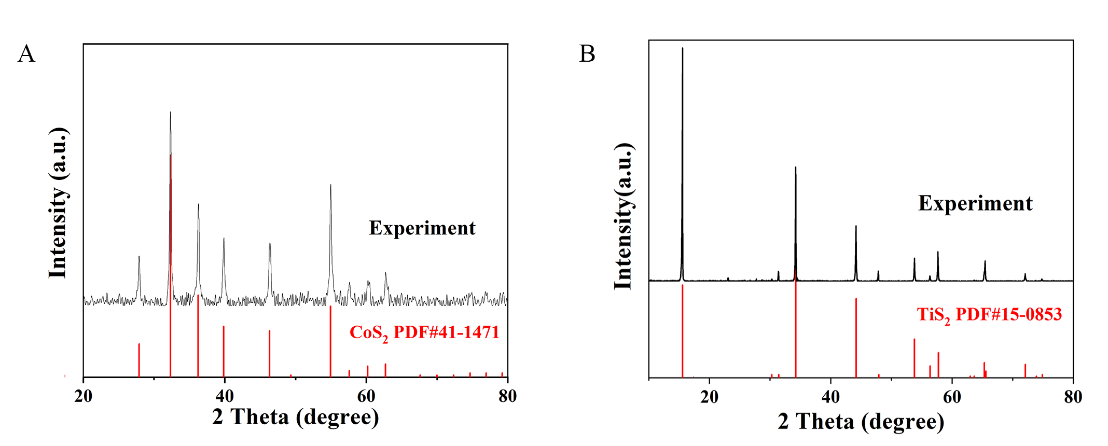


**FIGURE S1 |** XRD patterns of CoS_2_ **(A)** and TiS_2_ (**B**).


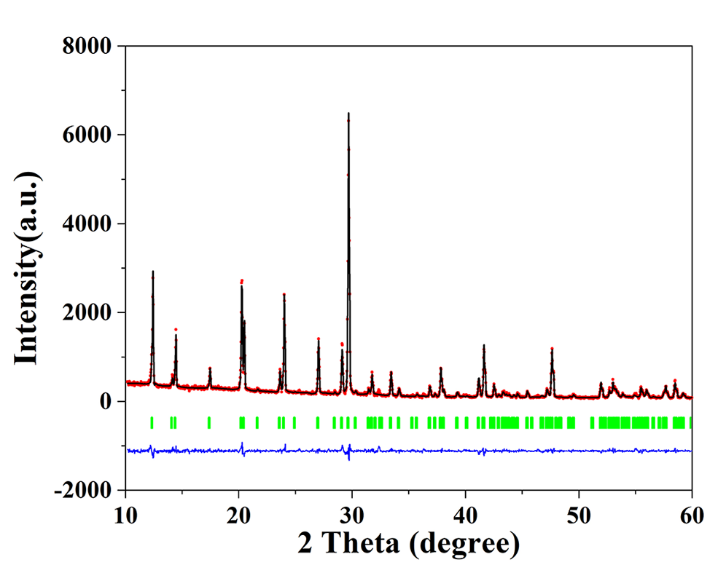


**FIGURE S2 |** Rietveld refinement of 1% Co^4+^ doped Li_10.35_Si_1.35_P_1.65_S_12_.


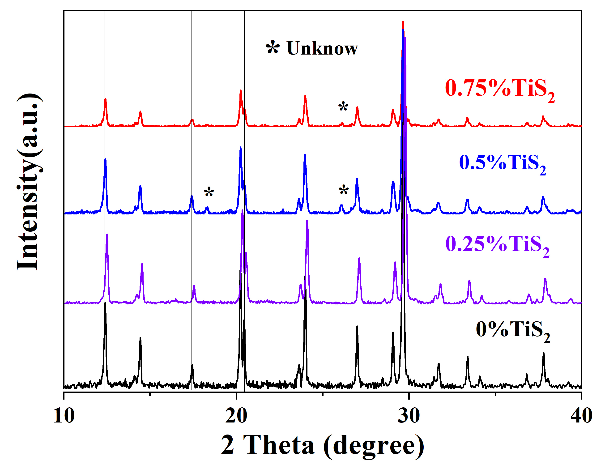


**FIGURE S3 |** XRD pattern of Ti^4+^ doped Li_10.35_Si_1.35_P_1.65_S_12_.


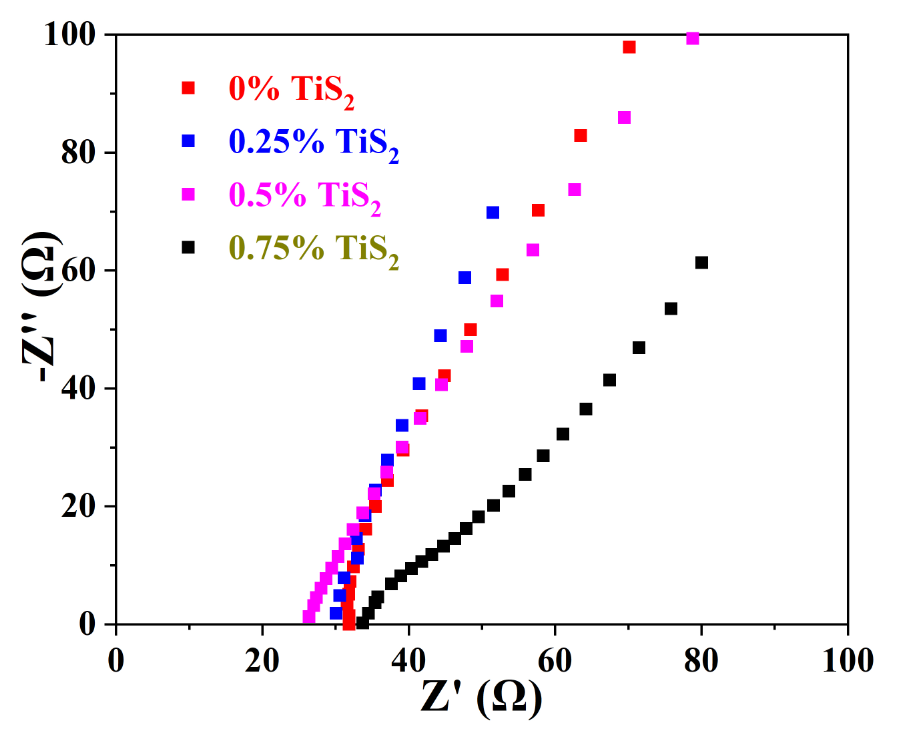


**FIGURE S4 |** Nyquist plots of Ti^4+^ doped Li_10.35_Si_1.35-x_Ti_x_P_1.65_S_12_.


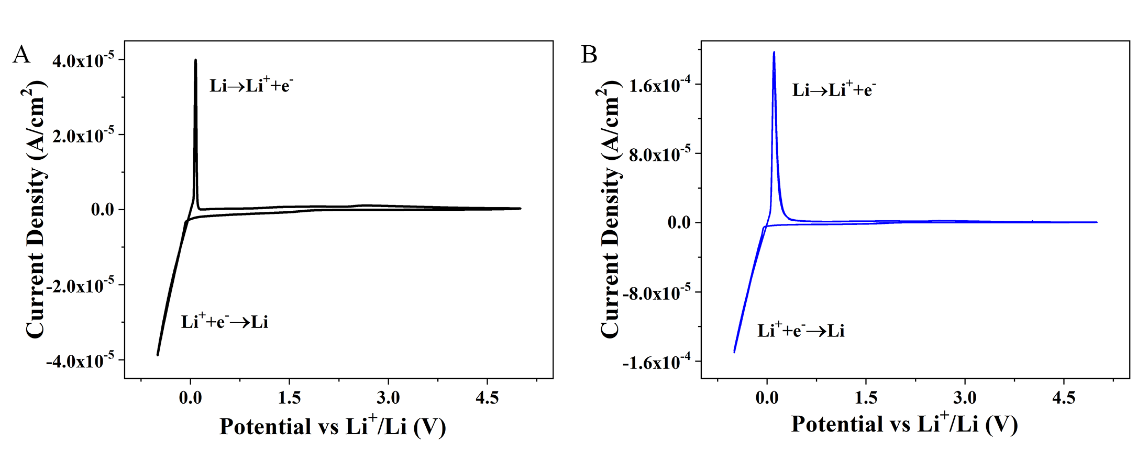


**FIGURE S5 |** CV curve of 0.5% **(A)** and 2% **(B)** Co^4+^ doped Li_10.35_Si_1.35_P_1.65_S_12_.


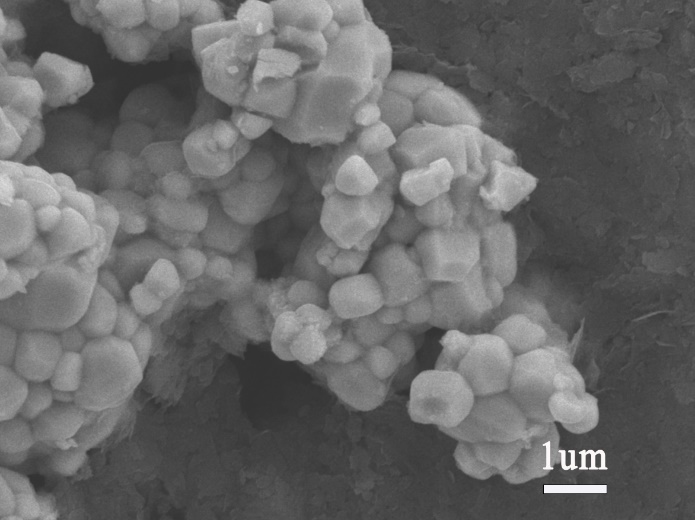


**FIGURE S6 |** SEM image of CoSe_2_.


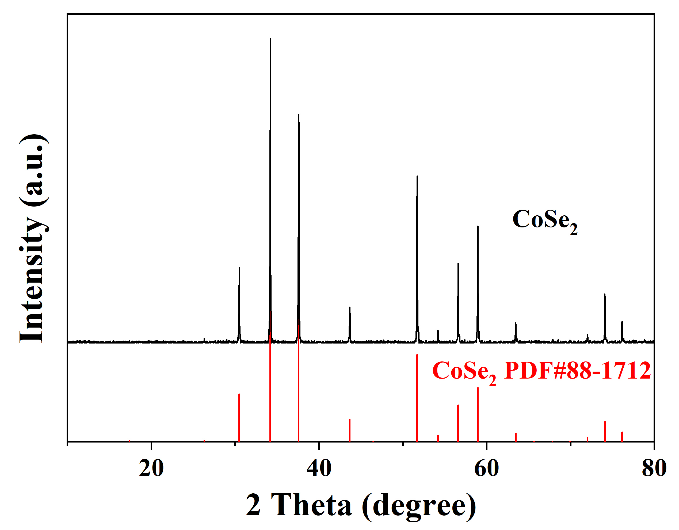


**FIGURE S7 |** XRD pattern of CoSe_2_.


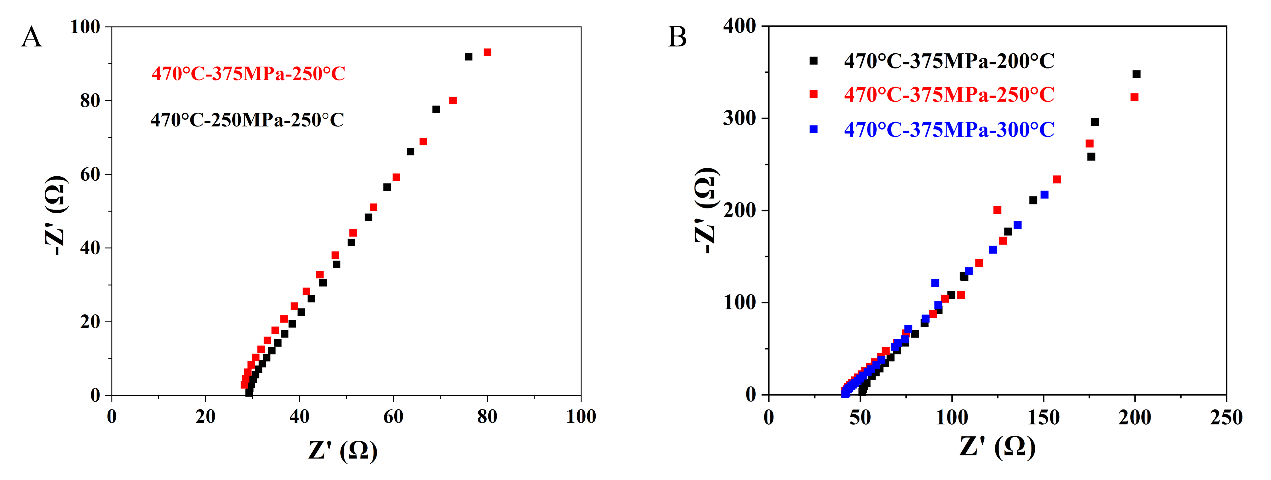


**Figure S8|**The Nyquist plots of Li_10.35_Si_1.35_P_1.65_S_12_ (470 ℃) tested under different (A) pressure and (B) temperature.


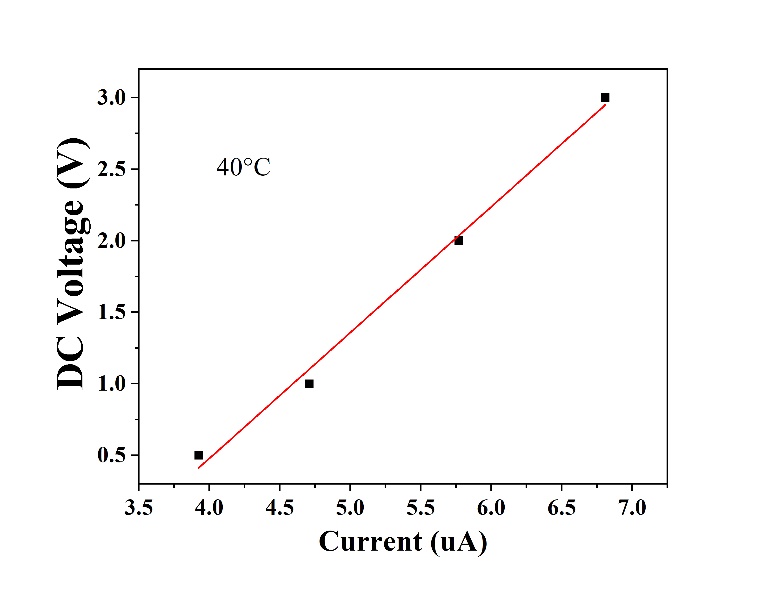


**Figure S9|**Voltage-current diagram of 1% CoSe_2_-doped electrolyte at 40 °C

**TABLE S1 |** The testing parameters of Li_10.35_Si_1.35_P_1.65_S_12_ synthesized at different temperatures.

| Synthetic temperature（^o^C） | 400 | 450 | 470 | 480 | 500 |
| --- | --- | --- | --- | --- | --- |
| Electrolyte quality（mg） | 200 | 200 | 200 | 100 | 100 |
| Electrolyte thickness（mm） | 1.06 | 1.34 | 1.24 | 0.344 | 0.36 |
| Impedance（Ω） | 44.7 | 55 | 36.8 | 16.7 | 18.3 |
| Ionic conductivity（mS/cm） | 3.02 | 3.10 | 4.28 | 2.62 | 2.51 |

**TABLE S2 |** Cell parameters of 1% Co^4+^ doped electrolyte.

| Atom | site | g | x | y | z | B |
| --- | --- | --- | --- | --- | --- | --- |
| Li(1) | 16h | 0.488 | 0.52034(0) | 0.03911(0) | 0.96540(0) | 1.908(0) |
| Li(2) | 4d | 1 | 0.25000(0) | 0.25000(0) | 0.69520(0) | 8.658(0) |
| Li(3) | 8f | 0.716 | 0.48562(0) | 0.98562(0) | 0.75000(0) | 3.506(0) |
| Li(4) | 4c | 0.791 | 0.25000(0) | 0.75000(0) | 0.98533(0) | 5.087(0) |
| Si | 4d | 0.63824 | 0.25000(0) | 0.25000(0) | 0.43900(69) | 3.118(284) |
| P(1) | 4d | 0.325 | 0.25000(0) | 0.25000(0) | 0.43900(69) | 3.118(284) |
| P(2) | 2b | 1 | 0.25000(0) | 0.75000(0) | 0.25000(0) | 3.589(423) |
| S(1) | 8g | 1 | 0.25000(0) | 0.93880(42) | 0.15797(40) | 4.212(203) |
| S(2) | 8g | 1 | 0.25000(0) | 0.04798(46) | 0.84804(37) | 2.586(203) |
| S(3) | 8g | 1 | 0.25000(0) | 0.44599(50) | 0.54051(35) | 2.353(214) |
| Co | 4d | 0.03676 | 0.25000(0) | 0.25000(0) | 0.43900(69) | 3.118(284) |

Notes：Crystal Cell：Tetragonal Crystal System, P42/nmc(137); a= 8.67316(5) Å, c=12.53313(8) Å, V=942.788(0.07) Å^3^; R_wp_=8.21%, R_p_=6.11%, R_exp_=6.10%, S=R_wp_/R_exp_=1.346.

**TABLE S3 |** The testing parameters Co^4+^ doped Li_10.35_Si_1.35_P_1.65_S_12_.

| Doping ratio（%） | 0 | 0.5 | 1 | 2 | 3 |
| --- | --- | --- | --- | --- | --- |
| Electrolyte quality（mg） | 200 | 200 | 200 | 200 | 200 |
| Electrolyte thickness（mm） | 1.24 | 1.11 | 1.26 | 1.15 | 1.24 |
| Impedance（Ω） | 31.9 | 22.67 | 23.22 | 22.17 | 32.69 |
| Ionic conductivity（mS/cm） | 4.95 | 6.24 | 6.91 | 6.60 | 4.83 |

**TABLE S4 |** Cell parameters of 1% CoSe_2_-doped electrolyte.

| Atom | site | g | x | y | z | B |
| --- | --- | --- | --- | --- | --- | --- |
| Li(1) | 16h | 0.488 | 0.49220(0) | 0.03715(0) | 0.96293(0) | 3.030(0) |
| Li(2) | 4d | 1 | 0.25000(0) | 0.25000(0) | 0.68565(0) | 10.056(0) |
| Li(3) | 8f | 0.716 | 0.46183(0) | 0.96183(0) | 0.75000(0) | 12.228(0) |
| Li(4) | 4c | 0.791 | 0.25000(0) | 0.75000(0) | 0.98613(0) | 6.947(0) |
| Si | 4d | 0.63824 | 0.25000(0) | 0.25000(0) | 0.43738(42) | 2.839(126) |
| P(1) | 4d | 0.325 | 0.25000(0) | 0.25000(0) | 0.43738(42) | 2.839(126) |
| P(2) | 2b | 1 | 0.25000(0) | 0.75000(0) | 0.25000(0) | 3.169(195) |
| S(1) | 8g | 0.96324 | 0.25000(0) | 0.94164(29) | 0.15733(27) | 4.665(89) |
| S(2) | 8g | 1 | 0.25000(0) | 0.04586(29) | 0.84723(22) | 2.238(71) |
| S(3) | 8g | 1 | 0.25000(0) | 0.44652(33) | 0.54207(22) | 2.602(77) |
| Se | 8g | 0.03676 | 0.25000(0) | 0.94164(29) | 0.15733(27) | 4.665(89) |
| Co | 4d | 0.03676 | 0.25000(0) | 0.25000(0) | 0.43738(42) | 2.839(126) |

Notes：Crystal Cell：Tetragonal Crystal System, P42/nmc(137), a=8.6772(1) Å, c=12.5353(4) Å, V=943.835(0.047) Å^3^, R_wp_=8.27%, R_p_=6.29%, R_exp_=5.92%, S=R_wp_/R_exp_=1.397.

**TABLE S5 |** Ionic conductivity data for previous studies

| Sample | Electrolyte Composition | Ionic Conductivity  (mS·cm^-1^) |
| --- | --- | --- |
| 1(Trevey J E et al.,2010) | Li_3.05_Ge_0.05_P_0.95_S_3.9_Se_0.1_(LGPS) | 1.4 |
| 2(Amaresh S et al.,2014) | Li_9.7_Al_0.3_Ge_0.7_P_2_S_12_ (LGPS) | 1.7 |
| 3(Z Liu et al.,2014) | Li_2.5_Ge_0.175_Ga_0.025_Se_0.80_PS(LGPS) | 1.5 |
| 4(Yang K et al.,2016) | Li_10_Ge_0.6_Sn_0.4_P_2_S_11.2_Se_0.8_(LGPS) | 2.75(1.8) |
| 5(Inagaki M et al.,2019) | Li_9.81_Sn_0.81_P_2.19_S_12_ (LSnPS) | 5.0 |
| 6(Bai Y et al.,2019) | Li_9.54_Si_1.74_P_1.44_S_11.7_I_0.3_ (LSiPS) | 1.35 |
| 7(Kwang-Hyun Kim et al.,2019) | Li_10_SiP_2_S_11.3_O_0.7_(LSiPS) | 3.1(1.6) |
| This work | 1% CoSe_2_-doped (LSiPS) | 6.07(4.28) |
| This work | 1% CoS_2_-doped (LSiPS) | 6.91(4.28) |

Trevey, J. E. , Jung, Y. S. , and Lee, S. H. . (2010). Preparation of Li2S-GeSe2-P2S5 electrolytes by a single step ball milling for all-solid-state lithium secondary batteries. *Journal of Power Sources*, 195(15), 4984-4989. doi: 10.1016/j.jpowsour.2010.02.042

Amaresh, S. , Karthikeyan, K. , Kim, K. J. , Lee, Y. G. , and Lee, Y. S. . (2014). Aluminum based sulfide solid lithium ionic conductors for all solid state batteries. *Nanoscale*, 6. doi: 10.1039/c4nr00804a

Z Liu, Y Tang, Y Wang, and F Huang. (2014). High performance Li2S–P2S5 solid electrolyte induced by selenide. *Journal of Power Sources*, 260(aug.15), 264-267. doi: 10.1016/j.jpowsour.2014.03.036

Yang, K. , Dong, J. , Zhang, L. , Li, Y. , and Wang, L. . (2016). Dual doping: an effective method to enhance the electrochemical properties of Li10GeP2S12‐based solid electrolytes. *Journal of the American Ceramic Society*, 98(12), 3831-3835. doi: 10.1111/jace.13800

Inagaki, M. , Suzuki, K. , Hori, S. , Yoshino, K. , and Kanno, R. . (2019). Conduction mechanism of LI10GEP2S12-type lithium superionic conductors in a Li-Sn-Si-P-S system. *Chemistry of Materials*, 31(9). doi:10.1021/acs.chemmater.9b00743

Bai, Y. , Zhao, Y. , Li, W. , Meng, L. , and Chen, G. . (2019). The new insight for solid sulfide electrolytes LSiPSI by using Si/P/S as the raw materials and I doping. *ACS Sustainable Chemistry and Engineering*, 7(15). doi:10.1021/acssuschemeng.9b01937

Kwang-Hyun Kim. , and Steve W. Martin. .(2019) Structures and Properties of Oxygen-Substituted Li10SiP2S12–xOx Solid-State Electrolytes. *Chemistry of Materials*, 31(11). doi: Chemistry of Materials
